# Evaluation of an Advanced Care at Home Pilot Program

**DOI:** 10.1001/jamanetworkopen.2025.10617

**Published:** 2025-05-09

**Authors:** Laura C. Myers, Martin Elliott, Gina Rinetti-Vargas, Phyllis Stark, Patricia Kipnis, Vivian Reyes, Vincent Liu

**Affiliations:** 1 Division of Research, The Permanente Medical Group, Oakland, California; 2 Kaiser Foundation Hospital and Health Plan, Oakland, California

## Abstract

This quality improvement study evaluates length of stay and health care use outcomes associated with an advanced care at home program.

## Introduction

Traditionally, acute care is delivered in hospitals, but hospitals are strained, especially during influenza season. A randomized clinical trial^1^ found that patients treated in the home vs the hospital underwent fewer tests, with a lower rate of 30-day readmission. We rigorously evaluated the advanced care at home (ACAH) program in Kaiser Permanente Northern California (KPNC).

## Methods

The KPNC Research Determination Office determined this quality improvement study to be exempt from institutional review board approval and informed consent. We followed the SQUIRE reporting guideline.

We previously described ACAH and identification of comparators.^2^ Briefly, ACAH provides high-quality care to adults with acute illness at home, leveraging telehealth monitoring and in-home intravenous antibiotics, phlebotomy, mobile imaging, and clinician assessment (eMethods in Supplement 1). ACAH was available September 1, 2020, to June 8, 2022, in Vacaville, Vallejo, and Walnut Creek hospitals if the program could meet patient needs. ACAH contained acute and restorative (providing intensive postdischarge home health care) phases. Comparators were identified electronically (September 1, 2021, to June 8, 2022), screened by electronic health record review and telephoned within 1 week of discharge from 1 of 18 nonparticipating hospitals.

Outcomes were (1) length of stay (LOS), (2) unplanned escalation to the emergency department (ED) or hospitalization during the acute phase (for ACAH only), and (3) ED treatment and release or hospitalization within 30 days after the acute phase. Missing values (<5%) for Laboratory-Based Acute Physiology Score, version 2 (LAPS2) and Comorbidity Point Score, version 2 (COPS2) were imputed to each group median. We performed linear or logistic regression, as appropriate, adjusting for age, sex, acute severity of illness (LAPS2^3^), comorbidity burden (COPS2^4^), COVID-19 status, and acute care visits within the 12 months prior. ACAH LOS included time spent in the ED, general ward, and acute and restorative phases of ACAH (specified where appropriate).

Subgroup analyses included (1) 146 ACAH patients September 1, 2021, to June 8, 2022, because ACAH was matured and comparators were contemporaneously enrolling and (2) 196 ACAH patients enrolled from the ward not ED. We used SAS version 9.4 (SAS Institute), and 2-tailed tests of significance at *P* < .05.

## Results

Among 226 ACAH patients (median [IQR] age, 57.5 [44.0-65.0] years; 57.5% male; 13.3% Asian, 9.7% Black, 15.5% Hispanic, and 59.7% White), with 0 losses to follow-up or deaths, and 715 comparators (median [IQR] age, 56.0 [42.0-67.0] years; 49.5% male; 11.5% Asian, 6.7% Black, 20.6% Hispanic, and 55.7% White), age was similar despite fewer Medicare Advantage ACAH patients (Table 1). ACAH enrolled a diverse population with higher median (IQR) illness severity (LAPS2, 67.0 [49.0-82.2] vs 59.0 [42.0-75.0]; *P* < .001) and comorbidity burden (COPS2, 16.0 [10.0-40.0] vs 10.0 [10.0-23.0]; *P* < .001) (Table 1). There were no differences in COVID-19 diagnosis or previous acute care use.

**Table 1.** Baseline Patient Characteristics

Characteristic	Patients, No. (%) (N = 941)		*P* value
	ACAH (n = 226)	Comparator (n = 715)	
Age, median (IQR), y^a^	57.5 (44.0-65.0)	56.0 (42.0-67.0)	.21
Sex			
Male	130 (57.5)	354 (49.5)	.04
Female	96 (42.5)	361 (50.5)	
Medicare Advantage	48 (21.2)	208 (29.1)	.03
Self-reported race and ethnicity^b^			
Asian	30 (13.3)	82 (11.5)	.03
Black or African American	22 (9.7)	48 (6.7)	
Hispanic or Latino	35 (15.5)	147 (20.6)	
White	135 (59.7)	398 (55.7)	
Other^c^	4 (1.8)	40 (5.6)	
LAPS2, median (IQR)^d^	67.0 (49.0-82.2)	59.0 (42.0-75.0)	<.001
COPS2, median (IQR)^e^	16.0 (10.0-40.0)	10.0 (10.0-23.0)	<.001
ED visit in 12 mo prior to admission	113 (50.0)	303 (42.4)	.05
Nonelective hospitalization in 12 mo prior to admission	49 (21.7)	127 (17.8)	.22
COVID-19–related admission	62 (27.4)	185 (25.9)	.64

Abbreviations: ACAH, advanced care at home; COPS2, Comorbidity Point Score, version 2; ED, emergency department; LAPS2, Laboratory-based Acute Physiology Score, version 2.

^a^ Median age in the ACAH group is increased compared to authors’ first study describing the derivation of the controls^2^ because more Medicare Advantage patients were enrolled (48 of 226 patients [21.2%] vs 3 of 126 patients [2.4%]).

^b^ Race and ethnicity are from self-report from the electronic health record and were included to report study population diversity.

^c^ Other race and ethnicity includes unknown, multiracial or multiethnic, or not listed in the other categories.

^d^ LAPS2 (range, 0-414; higher scores indicate increasing physiologic derangement) is assigned based on a patient’s worst vital signs, pulse oximetry, neurological status, and 16 laboratory test results in the preceding 72 hours. The univariate association of an admission LAPS2 with 30-day mortality was assessed (0-59: 1.0%; 60-109: 5.0%; ≥110: 13.7%).

^e^ COPS2 (range 0-1010; higher scores indicate increasing comorbidity burden) is assigned based on all diagnoses incurred by a patient in the 12 months preceding the index hospitalization. The univariate association of COPS2 with 30-day mortality was assessed (0-39: 1.7%; 40-64: 5.2%; ≥65: 9.0%).

ACAH had longer acute and acute with restorative LOS (Table 2) and 12 unplanned escalations (5.3%) during the acute phase. There was no difference in ED visit or hospitalization within 30 days from the end of the acute phase between ACAH and comparator groups (13.7% vs 14.1%; adjusted odds ratio, 0.88 [95% CI, 0.55-1.39]). Subgroup analyses results were nonsignificant. More than 90% of comparators confirmed interest in ACAH if available.

**Table 2.** LOS and Health Care Use by Study Group

Outcome	ACAH (n = 226)	Comparator (n = 715)	*P* value	Effect size (95% CI)
Index LOS, median (IQR), h				
Acute phase^a^	122.4 (96.7-167.2)	69.1 (49.3-98.3)	<.001	aRR = 2.11 (1.93-2.30)
Acute and restorative phases^b^	183.0 (135.3-265.5)	69.1 (49.3-98.3)	<.001	aRR = 3.20 (2.95-3.50)
Health care use, No. (%)				
Unplanned escalation during acute phase	12 (5.3)	NA	NA	NA
ED visit or hospitalization	31 (13.7)^c^	101 (14.1)	.57	aOR = 0.88 (0.55-1.39)

Abbreviations: ACAH, advanced care at home; aOR, adjusted odds ratio; aRR, adjusted risk ratio; LOS, length of stay; NA, not applicable.

^a^ Only acute phase counted for both groups.

^b^ For this analysis, LOS included time spent in acute and restorative phases for ACAH patients. The restorative phase is an integral part of the program for all patients enrolled and includes intensive physical therapy and medication management, which is more frequent and intensive than typical home care services. For comparators, LOS was calculated until the point that patients traditionally were discharged from the brick-and-mortar hospital.

^c^ Of 31 events, 18 events occurred when patients were in the restorative phase.

## Discussion

In this quality improvement study, ACAH patients had lower odds of acute care visits after the restorative phase but no difference counting from the acute phase. At early stages, there was a low threshold for return to acute care, which sometimes occurred for routine tests (CT scans) or procedures (thoracentesis) that were not offered via ACAH at the time. This may indicate that restorative phase services provide benefit during a critical postacute window. Most comparators showed interest, boding well for further implementation despite increased LOS.

Our findings add to the literature because the initial home hospital trial was conducted before the COVID-19 pandemic.^1,5,6^ We successfully enrolled many patients with COVID-19, offering promise to offload hospitals during future influenza seasons. The diverse ACAH population, with many Medicare Advantage enrollees, speaks to program generalizability.

A limitation is unmeasured confounding, although it is reassuring that ACAH patients had higher levels of comorbidity burden and acute severity of illness. Nonsignificant sensitivity and subgroup results were likely due to a reduction in power. Future analyses will include cost.

## Notes

Supplement 1. **eMethods.** Description of Advanced Care at Home (ACAH) Program

Supplement 2.
Data Sharing Statement
