# Supplementary material for: Evaluation of an Advanced Care at Home Pilot Program
Source: JAMA Netw Open. 2025 May 9;8(5):e2510617. doi: 10.1001/jamanetworkopen.2025.10617 (PMC12065028; doi:10.1001/jamanetworkopen.2025.10617)
Supplement: Supplement 1. — eMethods. Description of Advanced Care at Home (ACAH) Program [file jamanetwopen-e2510617-s001.pdf]

Supplemental Online Content

Myers LC, Elliott M, Rinetti-Vargas G, et al. Evaluation of an advanced care at home pilot program. *JAMA Netw Open*. 2025;8(5):e2510617. doi:10.1001/jamanetworkopen.2025.10617

eMethods. Description of Advanced Care at Home (ACAH) Program

This supplemental material has been provided by the authors to give readers additional information about their work.

© 2025 Myers LC et al. *JAMA Network Open*.

eMethods. Description of Advanced Care at Home (ACAH) Program

The details of the ACAH program are fully described in Elliot et al, Perm Journal, 2023, which is cited in the references. Beyond what is described in the main part of the text, the most pertinent details are as follows:

ACAH leverages an integrated care team and telehealth technology to provide hospital-level services and interventions, adapted for effective delivery in the home setting. In-home services include skilled nursing care, such as intravenous therapies, medication administration, wound care, Kaiser Permanente laboratory testing, mobile imaging services (including x-ray, ultrasound, and electrocardiogram), video consultation with specialists, and specialized therapy services, such as physical, occupational, and speech language therapists

Patients could enter the program via 2 pathways, "Acute Substitution" where they were enrolled directly from the emergency department without ever going to the general medical ward, and "Early Discharge" where they were enrolled after going to the general medical ward. While Acute Substitution pathways prevented an additional step in transferring to the ward, it was not always feasible to enroll patients when they were physically located in the emergency department. Hence, the Early Discharge pathway provides a second mechanism to screen for eligibility and offload the inpatient census. The subgroup analysis described in the main text was performed because patients admitted via these pathways could have different outcomes (theoretically, patients who spend more time inpatient have more inpatient work up and monitoring prior to enrolling into ACAH).

ACAH consists of 2 phases: acute and restorative phases. The clinical team determines when the patient is eligible to transition between the acute and restorative phases. The acute phase of ACAH mirrors the acute care delivered in the brick-and-mortar hospital with daily in-person visits by clinical staff, whereas the restorative phase is intensive home care. As an example, for someone in heart failure, the restorative phase would include in-depth medication management (e.g., down-titration of diuretics with blood draws and close monitoring of weights/salt intake) and more intensive physical therapy services than what is typically offered via traditional post-brick-and-mortar home care services. In the restorative phase, clinical visits are reduced and occur on an as-needed basis. Outcomes were assessed from both timepoints (end of acute phase and end of acute+restorative phase) in order to differentiate the impact of restorative phase, which the operational team felt was a critical component of ACAH.

© 2025 Myers LC et al. *JAMA Network Open*.
