# Supplementary material for: Evaluation of an Advanced Care at Home Pilot Program
Source: JAMA Netw Open. 2025 May 9;8(5):e2510617. doi: 10.1001/jamanetworkopen.2025.10617 (PMC12065028; doi:10.1001/jamanetworkopen.2025.10617)
Supplement: Supplement 2. — Data Sharing Statement [file jamanetwopen-e2510617-s002.pdf]

Data Sharing Statement

Myers. Evaluation of an Advanced Care at Home Pilot Program. *JAMA Netw Open*. Published online May 9, 2025. doi:10.1001/jamanetworkopen.2025.10617

Data
Data available: No

Additional Information
Explanation for why data not available: The datasets generated and/or analyzed during the current study are not publicly available due to their being the property of Kaiser Foundation Health Plan, Inc., but are available to interested collaborators in the context of a formal collaboration approved by the Kaiser Permanente Northern California Institutional Review Board for the Protection of Human Subjects.
